# The Pineal Gland

**Published:** 1916-06

**Authors:** 


**The Pineal Gland** is considered an inert, residual structure, by W. E. Dandy (Southern Med. Jour., Mch.) from experiments at Johns Hopkins. Puppies from which it was removed developed normally.

